# Development and evaluation of a TaqMan MGB RT-PCR assay for detection of H5 and N8 subtype influenza virus

**DOI:** 10.1186/s12879-020-05277-z

**Published:** 2020-07-29

**Authors:** Fan Yang, Lihua Xu, Fumin Liu, Hangping Yao, Nanping Wu, Haibo Wu

**Affiliations:** 1 State Key Laboratory for Diagnosis and Treatment of Infectious Diseases, National Clinical Research Center for Infectious Diseases, National Medical for Infectious Diseases, Collaborative Innovation Center for Diagnosis and Treatment of Infectious Diseases, The First Affiliated Hospital, School of Medicine, Zhejiang University, Hangzhou, 310003 China; 2 Animal Husbandry and Veterinary Institute, Zhejiang Academy of Agricultural Science, Hangzhou, 310021 China

**Keywords:** Avian influenza virus, H5N8, Virus detection, Minor groove binder probes, Multiplex real-time RT-PCR

## Abstract

**Background:**

Highly pathogenic influenza A (H5N8) viruses have caused several worldwide outbreaks in birds and are of potential risk to humans. Thus, a specific, rapid and sensitive method for detection is urgently needed.

**Methods:**

In the present study, TaqMan minor groove binder probes and multiplex real-time RT-PCR primers were designed to target the H5 hemagglutinin and N8 neuraminidase genes. A total of 38 strains of avian influenza viruses and other viruses were selected to test the performance of the assay.

**Results:**

The results showed that only H5 and N8 avian influenza viruses yielded a positive signal, while all other subtypes avian influenza viruses and other viruses were negative. High specificity, repeatability, and sensitivity were achieved, with a detection limit of 10 copies per reaction.

**Conclusions:**

The developed assay could be a powerful tool for rapid detection of H5N8 influenza viruses in the future.

## Background

Highly pathogenic avian influenza (HPAI) viruses are a threat to humans and animals, and cause considerable economic damage. The first H5N1 HPAI virus was detected in 1996 in a domestic goose in Guangdong, China (Gs/GD lineage), and caused deaths in wild birds, poultry and humans, and has since spread to over 80 countries in Asia, Europe, Africa and North America [1]. Since 2008, HPAI subtypes H5N2, H5N6 and H5N8 carrying the genetic backbone of the Gs/GD lineage H5 clade 2.3.4 have been identified in poultry worldwide, especially in domestic ducks and other birds in live poultry markets, and these subtypes have subsequently evolved into different subclades including 2.3.4.4 [2–4].

In early 2014, reassortant clade 2.3.4.4 H5N8 HPAI virus caused outbreaks in poultry in South Korea [4], and by late 2014, it had spread to Japan, Russia and Europe, with multiple cases reported from wild birds, including apparently healthy birds [5–7]. Subsequently, HPAIV H5N8 virus spread from Asia to North America and caused an outbreak leading to heavy losses of poultry in commercial farms in 2014–15 [8, 9]. The reassortant HPAIV H5N2 was composed of Eurasian HPAIV H5N8 and North American lineage AIVs, causing several outbreaks in Canada and North America [10], and affecting 232 farms in 15 states and more than 50 million birds in 2015 in the US [11]. In 2016 and 2019, the HPAI H5N8 virus caused successive epidemics in Nigeria, Cameroon, Egypt, Saudi Arabia and Namibia [12–17]. An increasing number of reports indicate that HPAI H5N8 viruses continuously cause deaths in wild migratory birds and birds in live poultry markets [18–20].

For more effective prevention and control of H5N8 infection, the development of a rapid, sensitive and specific diagnostic assay is critical. Currently, viral culture is the most traditional method for influenza diagnosis, and is considered the gold standard. However, it is time-consuming and complicated, and requires a laboratory with bio-safety level 3 practices [21]. Reverse-transcription PCR (RT-PCR) is the most well-established molecular detection technology currently available to detect and/or type influenza viruses [22]. Real-time RT-PCR (RRT-PCR), developed from RT-PCR, can monitor the progress of reactions by detecting the fluorescence signal in real time, resulting in higher sensitivity, specificity and simplicity [23]. In the present study, we developed a TaqMan minor groove binder (MGB) RRT-PCR assay to detect H5N8 subtype avian influenza viruses (AIVs) rapidly and specifically.

## Method

Three pairs each of specific primers and corresponding probes targeting H5 hemagglutinin (HA) and N8 neuraminidase (NA) genes were designed based on the nucleotide sequences of H5-HA (H5N1, H5N2, H5N6 and H5N8) (2.3.2 and 2.3.4.4) and N8-NA (H2N8, H3N8, H5N8, H6N8 and H10N8) genes from 1998 to 2018, obtained from the GenBank database, using Primer Express software as described previously [24]. Finally, two optimal sets of primers and probes for H5-HA and N8-NA (Table 1 and Fig. S1) were chosen after numerous comparison experiments as described previously [25]. A total of 34 strains of AIVs (Table 2) were selected to test the performance of the assay. Newcastle disease virus (NDV), infectious bronchitis (IBV) and infectious bursal disease virus (IBDV) were also used to assess specificity. And a reference real-time RT-PCR was performed using an Influenza A Virus Real Time RT-PCR Kit (Liferiver, Shanghai, China) according to the manufacturer’s instructions [26].

**Table 1:** Primers and probes used in multiplex real-time RT-PCR assays

Name	Sequence (5′ → 3′)	Position	Product
H5 Forward primer	AATGGGACGTATGACTAC	1495–1512	142 bp
H5 Reverse primer	TTGCCAGTGYTAGGGAAC	1619–1636	
H5 Probe	FAM-CAATAGGAACTTAC-MGB	1570–1583	
N8 Forward primer	TGGGTCTTTCACTTTACCAG	1209–1228	131 bp
N8 Reverse primer	CTCCATCGTGCCATGACC	1364–1381	
N8 Probe	HEX-CATTGTRATGTGTG-MGB	1326–1339	

**Note:** Primers and probes were targeted to the conserved regions of the H5-HA and N8-NA genes, and the H5 gene-specific probe was labelled with FAM at the 5′ end, while the N8 gene-specific probe was labelled with HEX to allow specific detection of H5N8 AIVs in a single reaction. The specific primers and probes were designed based on the nucleotide sequences of 781 H5N8-HA genes (including high pathogenic and low pathogenic H5N8), obtained from the GenBank database, using Primer Express software. By in silico analysis of published H5N8 sequence data, the primers and probes of H5 and N8 could perfectly match the 94.36% (737/781) and 92.8% (725/781) sequences, respectively. And 250 H5-HA genes (H5N1, H5N2, H5N6 and H5N8) from currently circulating clades (2.3.2, and 2.3.4.4) were obtained from the GenBank database, and the results showed 95.2% (238/250) sequences matched

**Table 2:** Avian influenza viruses and other viruses assessed in this study

Virus	Gene accession number		Subtype	Ct values from real-time RT-PCR		Reference real-time RT-PCR
	HA	NA		H5	N8	
A/duck/Zhejiang/D1/2013(H1N2)	KY971115.1	KY971171.1	H1N2	No Ct	No Ct	24.97
A/chicken/Zhejiang/2CP25/2014 (H1N3)	KY971138.1	KY971194.1	H1N3	No Ct	No Ct	14.85
A/duck/Zhejiang/473/2013(H1N4)	KF357774.1	KF357767.1	H1N4	No Ct	No Ct	27.33
A/chicken/Zhejiang/51043/2015(H1N9)	KY971141.1	KY971197.1	H1N9	No Ct	No Ct	32.94
A/duck/Zhejiang/465/2013(H2N7)	KF357792.1	KF357789.1	H2N7	No Ct	No Ct	24.85
A/duck/Zhejiang/6D10/2013(H2N8)	KX394376.1	KX394378.1	H2N8	No Ct	**26.66**	16.49
A/duck/Zhejiang/4613/2013(H3N2)	KF357818.1	KF357811.1	H3N2	No Ct	No Ct	16.58
A/duck/Zhejiang/5/2011(H3N3)	JX051229.1	JX051231.1	H3N3	No Ct	No Ct	33.00
A/duck/Zhejiang/D1–3/2013(H3N6)	KJ439856.1	KJ439878.1	H3N6	No Ct	No Ct	25.87
A/duck/Zhejiang/4812/2013(H3N8)	KF357821.1	KF357810.1	H3N8	No Ct	**20.72**	21.98
A/duck/Zhejiang/727145/2014(H4N2)	KT589211.1	KT589257.1	H4N2	No Ct	38.67	13.88
A/duck/Zhejiang/409/2013(H4N6)	KT589221.1	KT589267.1	H4N6	No Ct	No Ct	19.33
A/goose/Zhejiang/727098/2014(H5N1) (2.3.2)	KU042744.1	KU042802.1	H5N1	**16.93**	No Ct	10.83
A/duck/Zhejiang/6DK19/2013(H5N2) (2.3.4.4b)	KJ933377.1	KJ933379.1	H5N2	**19.22**	No Ct	32.11
A/duck/Zhejiang/6D2/2013(H5N6) (2.3.4.4b)	KJ807780.1	KJ807784.1	H5N6	**21.08**	No Ct	18.25
A/duck/Zhejiang/W24/2013(H5N8) (2.3.4.4b)	KJ476669.1	KJ476673.1	H5N8	**15.70**	**16.00**	26.01
A/duck/Zhejiang/6D18/2013(H5N8) (2.3.4.4b)	KJ476670.1	KJ476674.1	H5N8	**18.39**	**20.82**	18.92
A/duck/Zhejiang/925019/2014(H5N8) (2.3.4.4b)	KU042767.1	KU042825.1	H5N8	**27.82**	**25.29**	24.28
A/chicken/Zhejiang/1664/2017(H6N1)	MG063436.1	MG063440.1	H6N1	No Ct	No Ct	17.92
A/duck/Zhejiang/727038/2014(H6N2)	KT423148.1	KT423162.1	H6N2	No Ct	No Ct	23.29
A/chicken/Zhejiang/727018/2014(H6N6)	KU050771.1	KU050795.1	H6N6	No Ct	No Ct	29.02
A/duck/Zhejiang/DK16/2013(H7N3)	KC961629.1	KF042068.1	H7N3	No Ct	No Ct	18.36
A/chicken/Jiangxi/C25/2014(H7N7)	KM593186.1	KM593188.1	H7N7	No Ct	No Ct	16.29
A/chicken/Zhejiang/DTID-ZJU01/2013(H7N9)	KC899669.1	KC899671.1	H7N9	No Ct	40.21	25.91
A/chicken/Zhejiang/221/2016(H9N2)	KY056291.1	KY056305.1	H9N2	No Ct	39.79	17.22
A/duck/Zhejiang/6D20/2013(H10N2)	KP063197.1	KP063199.1	H10N2	No Ct	No Ct	22.87
A/chicken/Zhejiang/8615/2016(H10N3)	MG366506.1	MG366520	H10N3	No Ct	No Ct	20.23
A/chicken/Zhejiang/2CP8/2014(H10N7)	KP412451.1	KP412454.1	H10N7	No Ct	No Ct	16.98
A/chicken/Zhejiang/102615/2016(H10N8)	MG366509.1	MG366523.1	H10N8	No Ct	**17.52**	18.98
A/chicken/Zhejiang/102619/2016(H10N8)	MG366517.1	MG366524.1	H10N8	No Ct	**18.36**	18.00
A/chicken/Zhejiang/102622/2016(H10N8)	MG366511.1	MG366525.1	H10N8	No Ct	**26.02**	26.91
A/chicken/Zhejiang/121711/2016(H10N8)	MG366512.1	MG366526.1	H10N8	No Ct	**18.82**	12.99
A/duck/Zhejiang/727D2/2013(H11N3)	KX028817.1	KX028829.1	H11N3	No Ct	No Ct	18.29
A/duck/Zhejiang/71750/2013(H11N9)	KR864829.1	KR864831.1	H11N9	No Ct	No Ct	23.09
Newcastle disease virus (NDV)	–	–	La Sota	No Ct	No Ct	No Ct
Infectious bronchitis virus (IBV)	–	–	H120	No Ct	No Ct	No Ct
Infectious bursal disease virus (IBDV)	–	–	NF8	No Ct	No Ct	No Ct

**Note:** Assay results considered positive are indicated by Ct values in bold

By combining the sequences of H5 and N8, we developed a duplex RRT-PCR assay with two sets of primers and probes. Optimal concentrations of the two probes and primers were determined using the matrix method. H5 and N8 plasmids (pHW2000-H5 and pHW2000-N8 [27]) were serially diluted in 10-fold, with DNA ranging from 1 copy/mL to 1 × 10^5^ copies/mL and was detected with different amounts of forward primer, reverse primer and probe (Table S1 and Table S2). The optimal primer and probe concentration for the H5-HA primer pairs, N8-NA primer pairs, H5-HA probe, and N8-NA probe in the 20 μL RRT-PCR system was 250 nM in all cases. The RRT-PCR assay was performed in a 20 μL reaction mixture consisting of 10 μL 2 × One Step PCR Mix (Vazyme, China), 1 μL Enzyme Mix containing reverse transcription enzyme and DNA polymerase, 0.5 μL H5 forward primer (10 μM), 0.5 μL H5 reverse primer (10 μM), 0.5 μL H5 probe (10 μM), 0.5 μL N8 forward primer (10 μM), 0.5 μL N8 reverse primer (10 μM), 0.5 μL N8 probe (10 μM), 5 μL RNA sample, and 1 μL RNase-free water, as described previously [24]. Reactions were carried out in a C1000 Thermal Cycler Real-time RT-PCR instrument (Bio-Rad) and cycling parameters were 15 min at 55 °C, 5 min at 95 °C, 40 cycles of 5 s at 95 °C, and 34 s at 60 °C. No template control (NTC), positive control (H5N8 RNA) and negative control (water) reactions were also included, and data were analysed using a CFX96 Real-Time System.

The sensitivity of the RRT-PCR assay was determined for each reaction using 10-fold serial dilutions of H5 and N8 plasmids, with DNA ranging from 1 to 10^9^ copies per reaction [28]. To evaluate the clinical sensitivity and specificity of the RRT-PCR assay, six-week-old female BALB/c mice (*n* = 24) were anesthetized by isoflurane and inoculated intranasally with H5N8 virus in 0.05 mL phosphate buffered saline. Respiratory specimens and cloacal swab samples were harvested from mice at 3 days post-inoculation, and the mice were sacrificed with 5% isoflurane.

## Results

**Fig. 1:**
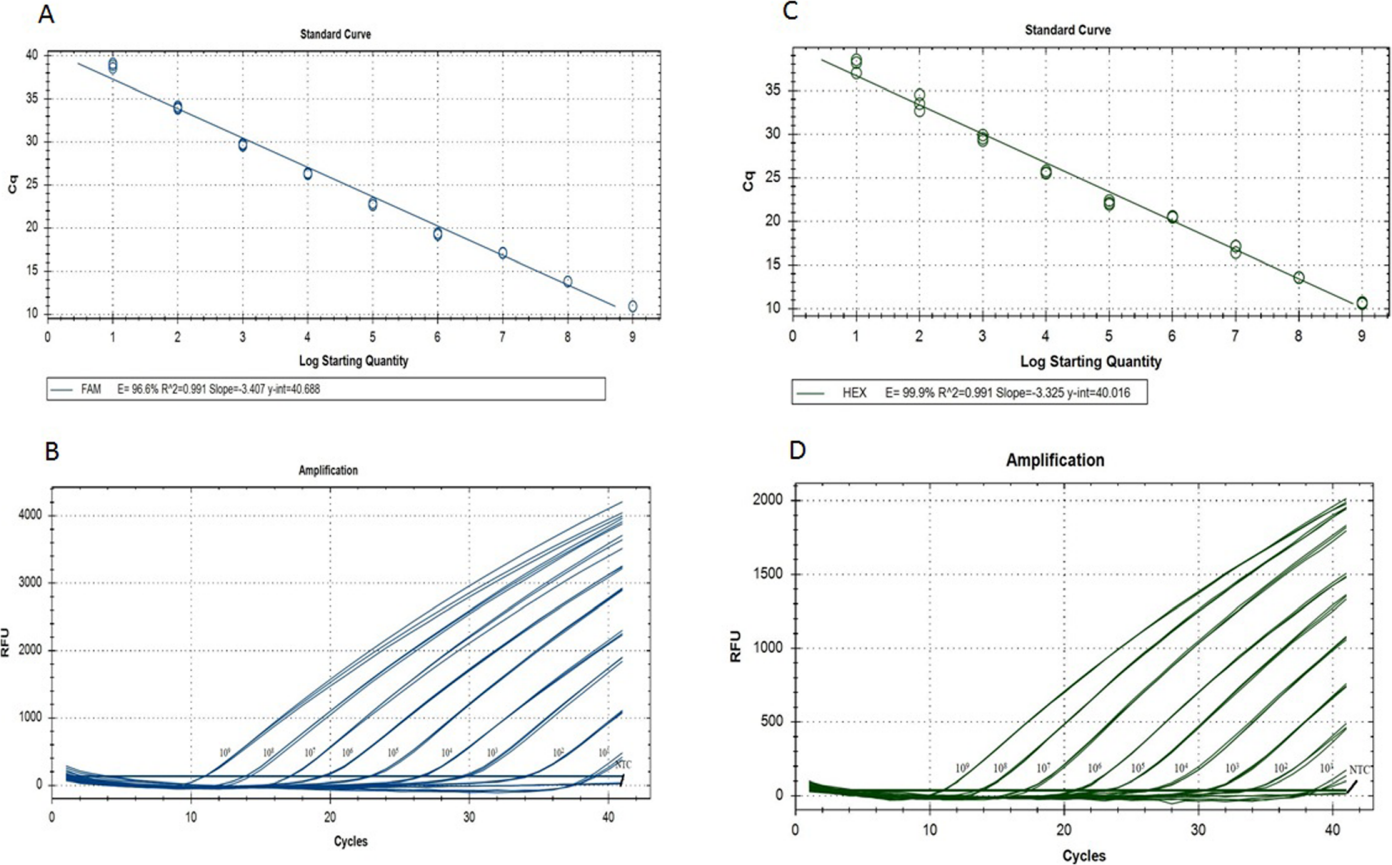
Amplification plots and standard curves of H5 (A and B) and N8 (C and D) assays. HA and NA gene sequences of pHW2000-H5 and pHW2000-N8 are from A/duck/Zhejiang/W24/ 2013(H5N8), an H5N8 AIV of clade 2.3.4.4 isolated in 2013

In this study, DNA plasmids were used for analytical sensitivity testing instead of RNA run-off transcripts as described previously [28]. The detection limit of the assay was 10 copies per reaction for both H5 and N8 genes. Standard curves for the two plasmids were generated by plotting their cycle threshold (Ct) values against DNA copy number, and both followed a linear correlation between 10 and 10^9^ copies of target DNA in each multiplex detection reaction (Fig. 1). Linear correlations of the standard curves of H5 and N8 were y = − 3.407x + 40.688 (efficiency = 96.6%, *R*^*2*^ = 0.991), and y = − 3.325x + 40.016 (efficiency = 99.9%, *R*^*2*^ = 0.991), respectively.

The diagnosis specificity of the assay was evaluated using the viruses listed in Table 2. The results showed that only H5 and N8 AIVs yielded a positive signal, while all other AIV subtypes and other viruses were negative.

Regarding reproducibility, inter-assays and intra-assays were analysed using different concentrations of plasmids as described previously [29]. The results of intra-assays (Table 3) and inter-assays (Table 4) revealed that the coefficients of variation (CV%) were all < 2%, suggesting our RRT-PCR method is highly reproducible [30].

**Table 3:** Intra-assay variation in multiplex detection of H5 and N8 avian influenza viruses

Plasmid copy number (per reaction)	Gene	Ct value in intra-assays			Mean ± SD	CV%
		1	2	3		
1 × 10^5^	H5	24.36	24.37	24.30	24.34 ± 0.03	0.13%
	N8	23.97	23.72	23.97	23.89 ± 0.12	0.49%
1 × 10^4^	H5	28.73	28.88	29.07	28.89 ± 0.14	0.48%
	N8	26.97	27.04	27.02	27.01 ± 0.03	0.11%
1 × 10^3^	H5	32.01	32.12	32.00	32.04 ± 0.05	0.17%
	N8	30.01	30.23	30.14	30.13 ± 0.09	0.30%
1 × 10^2^	H5	34.79	34.51	34.68	34.66 ± 0.12	0.33%
	N8	34.05	34.44	34.41	34.30 ± 0.18	0.52%
1 × 10^1^	H5	38.59	38.23	38.13	38.32 ± 0.20	0.52%
	N8	37.86	37.77	37.87	37.83 ± 0.04	0.12%
1 × 10^0^	H5	No Ct	No Ct	No Ct		
	N8	No Ct	No Ct	No Ct		

**Note**: Each concentration included three replicates on one plate. CV% (threshold = 3%) and Ct values for each concentration are shown

**Table 4:** Inter-assay variation in multiplex detection of H5 and N8 avian influenza viruses

Plasmid copy number (per reaction)	Gene	Ct value in intra-assays			Mean ± SD	CV%
		Day 1	Day 3	Day 5		
1 × 10^5^	H5	23.97	24.36	23.53	23.95 ± 0.34	1.42%
	N8	22.83	22.43	23.06	22.77 ± 0.26	1.14%
1 × 10^4^	H5	26.60	27.01	27.32	26.98 ± 0.29	1.09%
	N8	26.58	27.01	26.45	26.68 ± 0.24	0.90%
1 × 10^3^	H5	32.46	32.12	31.75	32.11 ± 0.29	0.90%
	N8	30.69	30.38	30.56	30.54 ± 0.13	0.42%
1 × 10^2^	H5	34.04	34.20	34.68	34.31 ± 0.27	0.79%
	N8	34.05	35.41	34.55	34.67 ± 0.56	1.62%
1 × 10^1^	H5	38.91	38.59	38.23	38.58 ± 0.28	0.72%
	N8	38.46	38.00	38.35	38.27 ± 0.20	0.51%
1 × 10^0^	H5	No Ct	No Ct	No Ct		
	N8	No Ct	No Ct	No Ct		

**Note**: We repeated multiplex RT-PCR on days 1, 3 and 5. CV% (threshold = 3%) and Ct values for each concentration are shown

**Fig. 2:**
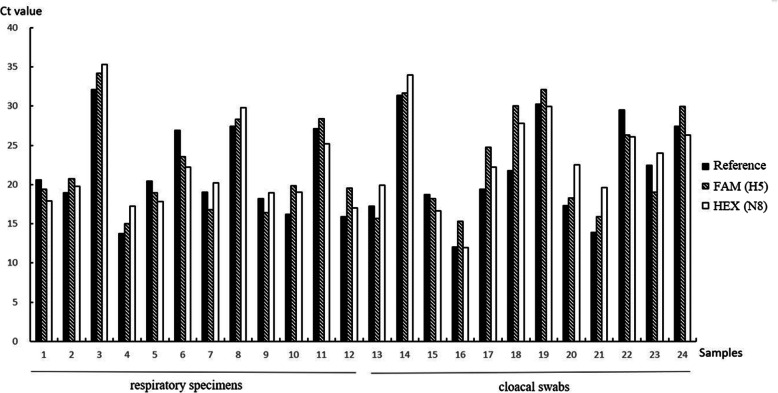
Detection of 24 laboratory-confirmed samples of H5N8 viruses by the multiplex real-time RT-PCR developed in this study. The Influenza Virus A&B Real-Time RT-PCR Kit was used in parallel as a reference. Ct values obtained from H5 and N8 assays for each sample are presented. Samples 1–12 are from respiratory specimens, and samples 13–24 are from cloacal swabs

Respiratory specimens and cloacal swab samples (*n* = 24) from mice infected with H5N8 [27] were collected and tested to evaluate the clinical sensitivity and specificity of the RRT-PCR assay. Concurrently, samples were also tested using an Influenza A Virus Real-Time RT-PCR Kit (Liferiver, China), and the results were used as a reference as described previously [24, 26]. Positive signals were obtained for all H5 and N8 samples, and the results were consistent with those obtained with the Influenza A Virus Real Time RT-PCR Kit (Fig. 2).

Additionally, a total of 148 cloacal swabs were collected from poultry in Zhejiang from 2013 to 2018 [31–33] and tested using both the RRT-PCR assay and virus isolation. The results of the RRT-PCR assay showed that there were 12 positive samples of H5N8 subtype AIVs, six positive samples of H5Nx subtype, and eight positive samples of HxN8 subtype, consistent with the results of virus isolation (Table 5).

**Table 5:** Comparison of the performance of multiplex PCR and virus isolation for 148 clinical specimens

Positive results	Virus isolation	Multiplex RRT-PCR
H5Nx^a^	6/148	6/148
HxN8^b^	8/148	8/148
H5N8	12/148	12/148
Total	26/148	26/148

^a^H5 subtype AIVs except H5N8 AIVs

^b^N8 subtype AIVs except H5N8 AIVs

## Discussion

Increasing evidence suggests that many subtypes of AIVs, such as H7N9, H10N8, H6N1, H9N2 and H7N7, are not only pathogenic for poultry, but they can also infect humans, and even cause death [34–36]. Historically, H5N1 and H7N9 AIVs have caused great economic losses and numerous deaths in humans [37, 38]. H5N8 HPAI has caused multiple disease outbreaks in poultry and wild birds, and has the potential to be transmitted from birds to humans. In view of the global threat posed by the H5N8 virus, an appropriate technology for timely detection and surveillance of this virus is required. A multiplex RRT-PCR assay for detecting H5N8 has been developed previously with a detection limit of 99.9 copies per reaction for the H5 gene and 15.9 copies per reaction for the N8 gene [39]. A riems influenza a typing array (RITA) was developed by duplex TaqMan reactions for detection and identification of 14 HA and 9 NA subtypes of AIVs, including H5 and N8 subtype AIVs. But no H5N8 AIVs were included in this study to verify the specificity of the RITA [40]. Additionally, a real-time PCR assay was developed to sensitively detect H5N8 of clade 2.3.4.4b HPAIVs, originating from European poultry and wild bird cases during 2016–2018 [41]. In the current study, the RRT-PCR was developed to detect the currently circulating H5N8 (including Eurasian lineage and North American lineage) by in silico analysis of published H5N8 sequence data. However, a total of 44 sequences (44/781) were incompletely covered by the primers and probe of H5, such as A/duck/Quang Ninh/19c511/2013 (H5N8), A/chicken/South Africa/499723/2018 (H5N8), and A/Duck/Egypt/F131/2019 (H5N8). In addition, a total of 56 sequences (56/781) were incompletely covered by the primers and probe of N8, such as A/common teal/Shanghai/PD1108–13/2013 (H5N8), A/duck/Taiwan/A3400/2015 (H5N8), and A/chicken/Belgium/807/2017 (H5N8). In silico mismatches do not necessarily translate into failure of detection in the wet assay. The capacity of the RRT-PCR developed in this study to cover the above strains should be further verified. In the present work, an efficient RRT-PCR assay was developed with a detection limit of 10 copies per reaction for both H5 and N8 genes via careful design and optimisation of primers and probes. Additionally, this assay performed well in the analysis of clinical samples.

## Conclusions

These results indicate that the duplex assay designed in this study is sufficiently sensitive and specific to be used for the detection of the H5N8 virus.

## Supplementary information

**Additional file 1: Table S1.** The optimal concentrations of H5 primers and probe. a. The most optimal concentrations of H5 primers and probe.

**Additional file 2:Table S2.** The optimal concentrations of N8 primers and probe. a. The most optimal concentrations of N8 primers and probe.

**Additional file 3:Figure S1.** Phylogenetic analysis (A and B) and sequence alignments (C and D) of the H5 and N8 genes of H5N8 influenza viruses. The tree was created by the maximum likelihood method and bootstrapped with 1000 replicates using the MEGA6 software version 6.0. The scale bar represents the distance unit between sequence pairs.

## Data Availability

The datasets supporting the conclusions of this article are contained within the article and its supporting files.

## Abbreviations

AIVs: Avian influenza viruses
Ct: Cycle threshold
CV%: Coefficients of variation
HA: Hemagglutinin
HPAI: Highly pathogenic avian influenza
IBDV: Infectious bursal disease virus
IBV: Infectious bronchitis virus
MGB: Minor groove binder
NA: Neuraminidase
NDV: Newcastle disease virus
NTC: No template control
RITA: Riems influenza a typing array
RRT-PCR: Real-time RT-PCR
RT-PCR: Reverse-transcription PCR
