# Supplementary material for: Development and evaluation of a TaqMan MGB RT-PCR assay for detection of H5 and N8 subtype influenza virus
Source: BMC Infect Dis. 2020 Jul 29;20:550. doi: 10.1186/s12879-020-05277-z (PMC7391517; doi:10.1186/s12879-020-05277-z)
Supplement: Supplementary file 1 — Additional file 1: Table S1. The optimal concentrations of H5 primers and probe. a. The most optimal concentrations of H5 primers and probe. [file 12879_2020_5277_MOESM1_ESM.docx]

**Table S1** The optimal concentrations of H5 primers and probe

| Amounts | 10 × 10^4^ | 10 × 10^3^ | 10 × 10^2^ | 10 × 10^1^ | 10 × 10^0^ |
| --- | --- | --- | --- | --- | --- |
| 0.1μL H5F, 0.1μL H5R, 0.1μL H5P | 37.23 | No Ct | No Ct | No Ct | No Ct |
| 0.1μL H5F, 0.1μL H5R, 0.5μL H5P | 36.85 | 38.99 | No Ct | No Ct | No Ct |
| 0.1μL H5F, 0.1μL H5R, 1μL H5P | 36.64 | 39.00 | No Ct | No Ct | No Ct |
| 0.5μL H5F, 0.5μL H5R, 0.1μL H5P | 36.50 | 37.89 | No Ct | No Ct | No Ct |
| 0.5μL H5F, 0.5μL H5R, 0.5μL H5P^a^ | 28.35 | 31.87 | 34.42 | 38.66 | No Ct |
| 0.5μL H5F, 0.5μL H5R, 1μL H5P | 27.98 | 31.84 | 34.98 | 34.72 | No Ct |
| 1μL H5F, 1μL H5R, 0.1μL H5P | 34.25 | 38.12 | No Ct | No Ct | No Ct |
| 1μL H5F, 1μL H5R, 0.5μL H5P | 27.22 | 30.69 | 37.11 | 38.74 | No Ct |
| 1μL H5F, 1μL H5R, 1μL H5P | 25.37 | 31.01 | 35.82 | 40.74 | No Ct |

a. The most optimal concentrations of H5 primers and probe.
