# Supplementary material for: Development and evaluation of a TaqMan MGB RT-PCR assay for detection of H5 and N8 subtype influenza virus
Source: BMC Infect Dis. 2020 Jul 29;20:550. doi: 10.1186/s12879-020-05277-z (PMC7391517; doi:10.1186/s12879-020-05277-z)
Supplement: Supplementary file 2 — Additional file 2:Table S2. The optimal concentrations of N8 primers and probe. a. The most optimal concentrations of N8 primers and probe. [file 12879_2020_5277_MOESM2_ESM.docx]

**Table S2** The optimal concentrations of N8 primers and probe

| Amounts | 10 × 10^4^ | 10 × 10^3^ | 10 × 10^2^ | 10 × 10^1^ | 10 × 10^0^ |
| --- | --- | --- | --- | --- | --- |
| 0.1μL N8F, 0.1μL N8R, 0.1μL N8P | 36.77 | No Ct | No Ct | No Ct | No Ct |
| 0.1μL N8F, 0.1μL N8R, 0.5μL N8P | 36.23 | 39.24 | No Ct | No Ct | No Ct |
| 0.1μL N8F, 0.1μL N8R, 1μL N8P | 37.03 | 38.92 | No Ct | No Ct | No Ct |
| 0.5μL N8F, 0.5μL N8R, 0.1μL N8P | 37.48 | 38.88 | No Ct | No Ct | No Ct |
| 0.5μL N8F, 0.5μL N8R, 0.5μL N8P^a^ | 26.85 | 30.77 | 34.01 | 37.55 | No Ct |
| 0.5μL N8F, 0.5μL N8R, 1μL N8P | 25.39 | 29.19 | 35.20 | 37.32 | No Ct |
| 1μL N8F, 1μL N8R, 0.1μL N8P | 36.03 | 39.11 | No Ct | No Ct | No Ct |
| 1μL N8F, 1μL N8R, 0.5μL N8P | 25.64 | 27.83 | 32.18 | 37.94 | No Ct |
| 1μL N8F, 1μL N8R, 1μL N8P | 25.36 | 29.97 | 35.86 | 38.47 | No Ct |

a. The most optimal concentrations of N8 primers and probe.
