# Supplementary figures and images for: Development and evaluation of a TaqMan MGB RT-PCR assay for detection of H5 and N8 subtype influenza virus
Source: BMC Infect Dis. 2020 Jul 29;20:550. doi: 10.1186/s12879-020-05277-z (PMC7391517; doi:10.1186/s12879-020-05277-z)

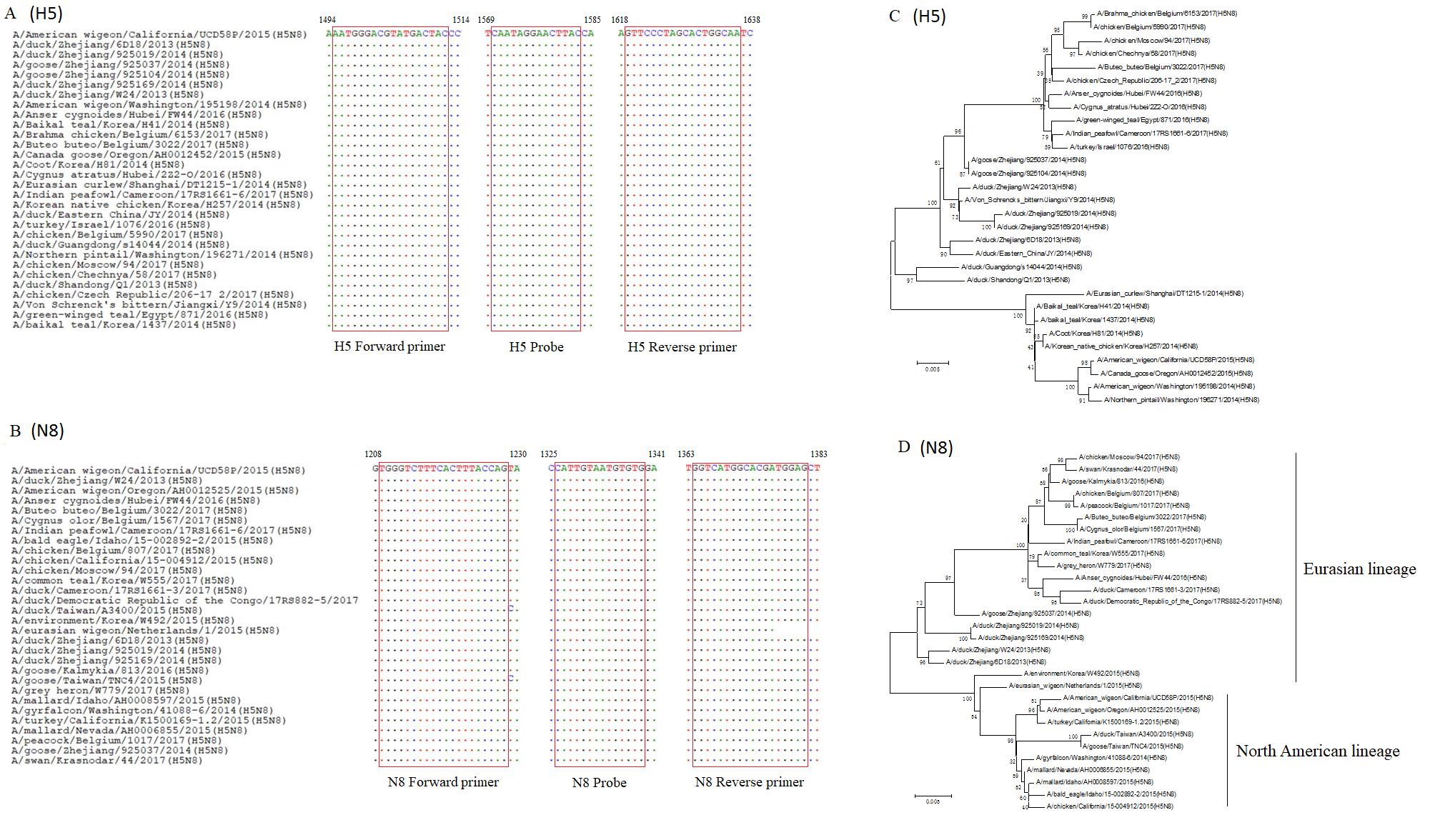

Supplement: Supplementary file 3 — Additional file 3:Figure S1. Phylogenetic analysis (A and B) and sequence alignments (C and D) of the H5 and N8 genes of H5N8 influenza viruses. The tree was created by the maximum likelihood method and bootstrapped with 1000 replicates using the MEGA6 software version 6.0. The scale bar represents the distance unit between sequence pairs. [file 12879_2020_5277_MOESM3_ESM.png]
